# Correlation between the Need for Cognitive Closure and Narrative Creativity in Secondary Education

**DOI:** 10.3390/ijerph18084333

**Published:** 2021-04-19

**Authors:** José Luis Ortega-Martín, Tatjana Portnova, Félix Zurita-Ortega, José Luis Ubago-Jiménez

**Affiliations:** 1 Department of Didactics of Language and Literature, University of Granada, 18071 Granada, Spain; ortegam@ugr.es (J.L.O.-M.); tportnova@ugr.es (T.P.); 2 Department of Didactics of Musical, Artistic and Corporal Expression, University of Granada, 18071 Granada, Spain; felixzo@ugr.es

**Keywords:** need for cognitive closure, narrative creativity, adolescents

## Abstract

(1) Background: The present study analyzed the need for cognitive closure and narrative creativity in adolescents. The aim was to demonstrate a strong relationship between narrative creativity and the need for cognitive closure. We analyzed a group of participants by applying a lie scale integrated with the Need for Closure Scale to detect potential relationships between students that entered the lie scale group (discarded) and those that were not discarded by exploring the following variables: gender, school type, group condition, and narrative creativity. (2) Methods: The instruments used were the Need for Closure Scale and the Test of Creative Imagination for Young People, PIC-J. Students of English as a foreign language in the 3rd year of secondary education from two schools were selected based on their availability to participate in the project. The students were aged 14 to 16 with a non-probabilistic sampling value of *N* = 117. (3) Results: Results show a negative correlation between narrative creativity and the need for cognitive closure. The need for cognitive closure is mainly manifested in two of its five dimensions: order and predictability. In addition, the group analysis of the lie scale revealed a higher tendency of male students to be less likely to respond truthfully. Meanwhile, the percentage of participants in the lie scale group was higher in rural schools. (4) Conclusions: In conclusion, students who do not belong to the lie scale group seem to have more creativity than students in the lie scale group, while students in the lie scale group have a lower final course grade than students in the non lie scale group.

## 1. Introduction

In the present work we analyzed the correlation between the need for cognitive closure and narrative creativity in adolescents. Additionally, the discarded answers of participants when applying the need for cognitive closure measure tool (lie scale) was also analyzed in order to establish their relationship to narrative creativity. Applying the lie scale criteria we created two groups: those students who discarded applying the lie scale and those not discarded (who answered the need for cognitive closure tool sincerely). Both groups were analyzed applying the variables of narrative creativity, gender, school type, and school class in order to find the difference between both groups. We expected that the condition of not being sincere when performing the test would be associated with one of the variables described.

Regarding the need for cognitive closure and creativity, the present work has as an antecedent several recent studies by Rocchi [1], Chirumbolo et al. [2], García-Ramírez and Hazir [3], García-Ramírez [4] and Sankaran et al. [5]. The studies have pointed out the interrelation between creativity and the need for cognitive closure. In this interrelation the creativity seems to be a possible tool to prevent the need for cognitive closure. Creativity mechanisms seem to help develop divergent thinking and, in this way, to reduce several individual and social phenomena associated. Unlike the studies mentioned above, we worked with adolescents in order to establish if the relationship between creativity and the need for cognitive closure can be detected in secondary school students (aged 14–16 years).

### 1.1. Need for Cognitive Closure

The need for cognitive closure (NFC) refers to the motivation of individuals to find and keep a definitive answer for a specific problem, which is the opposite of confusion, ambiguity and/or doubts [6,7]. This epistemic theory was developed by Webster and Kruglanski [8] and states that NFC is a latent variable that manifests itself through the following aspects: desire for predictability, preference for order and structure, disagreement with ambiguity or indecision, and closed-mindedness. NFC is the desire to have certain knowledge about a concept, while any type of ambiguity causes a state of discomfort [6,9]. A person with a high NFC level tends to get the information as quickly as possible and keep it unchanged for as long as possible.

This has a consequence of two main tendencies: a tendency of urgency to achieve concrete knowledge, and a tendency of permanence when this knowledge first “freezes” into the future. The knowledge acquired in the past remains permanent to ensure knowledge of the future. This can provoke persuasiveness before setting a constant opinion on a topic [10] or personal phenomena such as the inability to generate hypotheses or empathy [11]. Additionally, this may have the following consequences on an individual level: cognitive impatience, impulsivity, rigidity of thought, incapacity to change one’s mind, the formation of social prejudices or the formation of cultural stereotypes or stereotypes about people (on an individual level). At the group level, people with a high need for cognitive closure tend to search for consensus, they tend to show prejudice in a group of people and prefer homogeneous to heterogeneous groups. Furthermore, they are characterized by the tendency to develop an autocratic leadership over a democratic one within the decision-making structure in a group process [2]. People with high NFC tend to rely on initial information about concepts without being able to develop their own opinion about them. At the same time, they are less capable of empathy and communication skills [7]. A comprehensive study of the negative consequences can be found in Kruglanski and Fishman [11]; in the present study we underlined the primary negative aspects associated with high levels of NFC.

On the other hand, a very low level of NFC is not particularly positive either. The main consequences of NFC are preference for order and structure, discomfort with ambiguity, and decisiveness [8], which may be positively interpreted in certain contexts. As an example, Kruglanski and Fisher [11] highlight that NFC allows individuals to make a decision when forming knowledge that has important implications for social interaction. In the present study we mostly concentrated on the importance of preventing the high levels of NFC that may have several negative consequences as described above.

In contrast, people who have a high need for cognition which, unlike the need for cognitive closure, signifies the motivation and preference that people have towards the thinking process [12], are characterized by a skeptical approach towards prejudices and tend to reflect on the subject from different perspectives, avoiding a rigid opinion; they also perceive themselves to be more creative [13].

The need for cognitive closure should be detected and controlled from an early age in order to prevent the development of potentially negative consequences. As Chirumbolo et al. [2], García-Ramírez and Hazir [3] and García-Ramírez [4] worked with adults (the youngest level is in the study of García-Ramírez, where the participants are university students) when analyzing the interrelation between creativity and NFC, we wanted to assess the situation with secondary school students to detect the possible presence of higher levels and the potential relationship with creativity in younger age groups. In a school context it is important to deal with the aspects related to closed-mindedness as it affects students who still have not fully developed their personality and so negative aspects related to NFC, as mentioned above, may be aggravated in the future if not controlled. NFC should be one of the aspects controlled at the school level to prevent negative consequences that need to be treated afterwards.

A tool to measure NFC is the Need for Closure Scale (NCS) developed by Webster and Kruglanski [8]. This measures the general NFC scale and specific subscales of order, predictability, decisiveness, ambiguity and closed-mindedness. The tool has an incorporated lie scale in order to eliminate those answers that were not sincere. Usually the answers based on the lie scale are simply eliminated from the study as these are not valid. In the case of the present study, NFC was analyzed as well as creativity. Several studies establish a certain relationship between creativity and lying (see Section 1.5). These studies confirm the relationship between creativity and honesty/lying features, although there is a lack of uniformity of the results. In the present study, we paid analytic attention to the lie scale group with the purpose of establishing the possible relationship between creativity and lying.

### 1.2. Need for Cognitive Closure and Creativity

García-Ramírez [4] when analyzing the need for cognitive closure underlines that this very complex phenomenon depends on the attitudes, beliefs and previous experiences of the individual, relating to the different contexts in which they usually develop. He states it is not about avoiding the need for cognitive closure but about managing it throughout life.

Chirumbolo et al. [2] performed an experiment to establish the relationship between creativity and NFC. Participants in small groups consisted of people with high levels of NFC and others with low levels of NFC. The groups had to perform a task which consisted of creating advertisements. The results showed that the fluency of ideas, degree of elaboration and creativity (according to the judgments of independent evaluators) were lower in participants with high NFC. García-Ramírez and Hazir [3] carried out another study to detect the relationship between creativity and NFC and suggested that creativity is a tool for controlling cognitive closure “*in order to solve problems that arise during the learning process and in general during life*” (p. 18).

Sankaran et al. [5] also pointed out that the participants with high indexes of NFC had lower creativity (for the variables of fluency, originality, persistence and partially flexibility), while lower indexes of NFC corresponded to higher creativity indexes.

Kruglanski [7] highlights that high NFC in people entails several negative impacts on personality, which as a consequence is reflected in lower creativity. After analyzing several studies [1,14,15] that establish the relationship between creativity and NFC, the researcher concludes that high NFC trends lead to limiting the number of hypotheses generated and generating conventional ideas instead, which reduces the creativity of the individual when acting in a group. This shows that at the education stage higher levels of NFC may interfere with the learning process where divergent thinking is a tool to successful results and later at university level, a necessary research tool.

The three studies that Kruglanski mentions are based on the following: Rocchi [1] carries out two studies, one with American participants and the other with Italian students. During the study, the participants had to combine geometric elements in images, and their creativity indices were contrasted with their NFC levels, showing negative results between creativity and NFC. However, the study by Ward et al. [14] with American and Chinese participants, based on a similar research design (participants had to write down the first prototype associations for birds, fruits, trades, etc., instead of composing images), demonstrated a positive relationship between NFC and creativity. The studies by Chirumbolo et al. [15] focused on creating two groups of participants. One group composed of members with high NFC and the other, with low NFC. The participants had to create some product slogans. The results of this study suggest that there is a clear relationship between NFC and the ability to flow ideas, degree of elaboration, and creativity.

Wronska et al. [16] pointed to the importance of creativity in the psychological phenomena of people and highlighted the importance of creativity in the life of each person. Their study showed that participants high in NFC were less competent and felt more negative emotions when solving the divergent character tasks. They underline that in the education field, creativity may improve personal capacities of students.

We can observe that a high NFC seems to have negative consequences on personality and creativity. By finding the tools to control NFC, we can avoid the tendency for certain negative traits to develop in a person. As has been stated before, in education (especially in school) the objective of the teacher should not be purely to teach the subject, but also to understand that education includes the development of personal, social and psychological capacities that go hand in hand with the teaching and learning process. In the present study we have focused on adolescents in order to study the importance of NFC in the adolescent age group that has not been addressed in the antecedent studies.

### 1.3. Narrative Creativity

The studies regarding the interrelation between the need for cognitive closure and creativity resort to the creativity indexes. In the present work we will focus on narrative creativity indexes. Artola et al. [17] divided creativity into narrative creativity and figurative creativity where the sum of both gave the index of general creativity. In the present study the following definitions of different terms of creativity proposed by Artola et al. [17] are used. Narrative creativity is a measure of divergent thinking when it is applied to verbal-content problem solving. Figurative creativity is the level of an individual to create new associations and new combinations working in non-verbal contexts. General creativity is the estimation of the creative potential of the subject, of their ability to transform, combine and establish new relationships between the elements, generating their own ideas [17].

In the present study it was decided to focus only on narrative creativity as the participants were students of English as a foreign language (among language school courses this course was selected because of convenience as the teachers agreed to collaborate, allocating class hours for study). Additionally, this context was considered as mostly based on verbal tasks, which was especially important for the use of analysis related to the participants’ final notes from class, without having any figurative creativity context.

### 1.4. Lie Scale

The Need for Closure Scale [18] has an integrated lie scale. The lie scale is an inseparable element of the majority of tests where it is important to discard the answers of the quantitative tests that were not answered in a sincere way or paying attention to the task. Usually, the data obtained from the lie scale are deleted by the researchers as they are interpreted as unnecessary regarding the results of the study. A minimum quantity of examples can be found where the studies do pay attention to this group. As for example, when examining the Eysenck’s Personality Inventory (EPI) the lie scale results can be taken into account and compared with verbal IQ. Eysenck, Nias and Eysenck [19] interpreted the lie scale itself to conclude this rather than believe the propensity to lie may be the result of a lack of insight. Eysenck, Eysenck and Shaw [20] realized four experiments in order to discover the effects of instructions on lie scale results. This study is especially relevant for the present work as it concludes that the degree of motivation for dissimulation is positively correlated with the size of these correlations in the negative direction, which brings about the motivation as an aspect that affects the results regarding the lie scale in a test. As far as we know, no studies applying the Need for Closure Scale [7] analyze the discarded answers from the lie scale group.

### 1.5. Integrity and Creativity

The lie scale can also be interpreted in a more literal sense: those responses that were not sincere were detected by the lie scale. In the present study we interrelated the Need for Closure Scale results with narrative creativity, analyzing the lie scale group as a subgroup with results extracted from the Need for Closure Scale in order to find a relationship with narrative creativity indexes in adolescents. In this way, creativity enters into a possible relationship with integrity. Several studies exist that explore the relationship between creative personality and individual differences [21,22,23,24]. Generally, all of the authors agree that creativity is a complex phenomenon where cognitive, social, cultural and personal aspects interact. Here arises the difficulty of the studies related to creativity, as it cannot be studied separately from a lot of interacting complex variables that may also be involved in the creative process.

Most of the authors consider creativity as something generally positive [25]. Amongst the most cited positive impacts are those that impact divergent thinking [3,4,26], tolerance, flexibility, a wide range of interests [17], problem solving and the capacity to explore problems and generate ideas [27]. Generally, creativity is something positively promoted as something for the common wellbeing and interaction between people [28].

On the other hand, there is always a dark side. In this case, speaking about creativity and its relation to personal character features, we can also find studies that highlight the possible negative aspects of creative people. Thus, Beaussart, Andrews and Kaufman [25] underlined a recent concept that was found among the studies which is malevolent creativity. Malevolent creativity may be the case when the outcome of the creative process may be harmful or cruel for other people or for society. This can take root in a complex interaction of creativity with other character features we previously mention. A different (creative) result may also be a violation of the norms and habits.

The subject of integrity related to creativity is controversial in this field of studies. Regarding the honesty-humility variable, Silvia et al. [29], applied the HEXACO [30] model in their study to show that people with lower indexes of honesty-humility have higher creativity scores. Beaussart et al. [25] found a significant negative connection between self-reported integrity and creativity. Different results were obtained in the study of Sanz de Acedo and Sanz de Acedo [31]. They found a positive relationship between creativity and integrity-honesty. Gino and Ariely [32] detected that creative people frequently manipulate the results of their tests and concluded in their study that creativity could foster dishonesty. Walczyk et al. [33] reached the same conclusions in the field of fluency and lying (fluency, being one of the variables of creativity, enables the production of a lot of ideas and the use of fantasy that increase the cases of lying).

### 1.6. Objectives

The main aim of this study is to analyze the relationship of narrative creativity and the need for cognitive closure in adolescents. We intend to demonstrate that there is a close relationship between the narrative creativity and the need for cognitive closure at this age. 

Secondly, we intend to analyze the group of participants that discarded applying the lie scale and were integrated into the Need for Closure Scale. We aimed to detect the possible relationship between those students whose answers were entered in the lie scale group and those that were not discarded exploring the following variables: gender, school type, group condition (exploring if there is a difference among the six school classes that participated in the study), and narrative creativity. As the analysis of the lie scale group seemed not to be applied in previous need for cognitive closure studies, it is difficult to establish the hypothesis regarding this second objective.

## 2. Materials and Methods

### 2.1. Subjects and Design

The present study was held in 2019 in two secondary schools situated in the city of Granada (Spain). The participants were students of English as a foreign language enrolled in the 3rd grade of secondary education within two schools selected by the criteria of availability to participate in the project. We used non-probability sampling in our study and final numbers were 117 participants, aged from 14–16 years old. In relation to their nationality, they were: 111 Spanish, 2 Romanian, 1 American, 1 German and 2 Peruvian. Regarding their gender: 50 were male and 67 were female. One school was situated in a rural area and the other in an urban area.

### 2.2. Instruments and Variables

A questionnaire given to the students to complete (ad hoc) was used to compile socio-demographic information. Participants’ gender (categorized as either male or female) and age were recorded.

In order to evaluate the creativity of young people, we used the Test of Creative Imagination for Young People, PIC-J, developed by Artola et al. [17] as a scale. This test evaluates creativity through the use of imagination by individuals, and it is specifically designed for the Spanish population. It consists of four activities: three tasks to assess narrative creativity (expressed through words) and one to assess figurative creativity (expressed through drawings). The first task consists of writing sentences that come to mind to describe a picture included in the test. The second task consists of writing all the possible uses of a given object (rubber tube). In the third task the student is presented with an unlikely situation “what would happen if the ground suddenly becomes elastic” and he/she must give possible answers. The sum of the three previous tasks measure narrative creativity. The fourth and final task consists of complementing the lines presented in four squared spaces to turn them into a drawing. This task measures figurative creativity. The test has correction instructions designed to facilitate the interpretation of the answers obtained through the test. Giving points for each answer in order to obtain numeric results for fluency, flexibility and originality dimensions, the sum of the three dimensions gives the narrative/figurative creativity indexes. The sum of narrative and creativity indexes gives a general creativity score. In the present study we focused on narrative creativity only, as the context selected for the study was solely based on verbal tasks (English as a second language students).

The PIC-J instrument measures creativity by considering different variables such as the fluency of ideas, thinking flexibility, response originality and elaboration of answers, as well as the use of creative details such as color, shadows, movement, etc. It can take between 45 min and 1 h to fully implement. The PIC-J has been used with large and representative samples, showing a Cronbach alpha of 0.83 [34].

The Need for Closure Scale (NCS) was developed by Webster and Kruglanski [8] to assess the need for cognitive closure in subjects; the updated version was used for this study [18]. This scale is composed by 47 items answered on a Likert-type scale with six response options (“1 = strongly disagree” to “6 = strongly agree”), 11 items are reverse-scored across five subscales: order, predictability, decisiveness, ambiguity and closed-mindedness.

### 2.3. Procedure

Firstly, scientific research was carried out in order to gather information and clarify some of the current problematic situations in adolescent people. In order to complete the data collection processes, different schools that were selected via convenience sampling were contacted. Once permission was received from the schools, an information pack was developed that was targeted towards the students’ legal guardians so that the students could participate in the study while ensuring anonymity at all times. In both schools, the parents or legal guardians signed the corresponding authorization to carry out the study. Likewise, permission was obtained from the Territorial Delegation in Granada for Education, Culture and Sports to carry out the research. Researchers were present throughout the data collection processes in order to resolve any doubts arising during questionnaire completion. The present study complied with the ethical principles for research with human subjects established by the Declaration of Helsinki in 1975. All processes were also conducted under the supervision of the research ethics committee at the University of Granada (462/CEIH/2017).

### 2.4. Data Analysis

The statistical software SPSS 25.0 (IBM Corp, Armonk, NY, USA) was used to process the data, in order to establish the frequencies and mean of the basic descriptive analysis. Cronbach’s coefficient was used to determine the internal consistency of the instruments, establishing the reliability index at 95%.

## 3. Results

### 3.1. Narrative Creativity and NFC Factors

First of all, to analyze the variables related to NFC, we discarded those answers of the students whose lie scale was higher that 15 (*N* = 34). Non-valid answers (34 of 117), were analyzed separately, we will discuss them below.

Based on narrative creativity scores, all the students were divided in three quartiles: low, medium and high.

In Table 1 we observed the distribution of each group for the five factors of need for cognitive closure based on gender and narrative creativity (NC) quartiles. To analyze the relationship between narrative creativity and the need for cognitive closure factors, t-tests for two independent samples were applied. Taking into account the average scores for the five factors and comparing the low narrative creativity group (Q1) with the high creativity group (Q3), we detected that the differences between both groups were significant for the variables of order (*p* = 0.034) and predictability (*p* = 0.018). The comparison of the extreme groups (low Q1 and high Q3) followed the design of the previous study by García-Ramírez [4]. No statistically significant associations were found for the medium level (Q2) compared to the low (Q1) and high (Q3) levels.

When applying the analysis based on gender scores for the five factors, we detected that the results were not statistically significant (*p* > 0.05). This shows that gender is not interrelated with the NFC.

To check if there were differences in the NFC factors in different groups of students (six different classes participated in the project), a one-way ANOVA was performed to analyze whether the differences according to the group belonging were statistically significant. Statistically significant differences were found between participants’ gender and NFC dimensions of order (*p* = 0.000) and predictability (*p* = 0.004). 

### 3.2. Narrative Creativity and General NFC Score

To analyze the interrelation between narrative creativity and the need for cognitive closure, we divided the participants into three quartiles based on their scores of NFC. In Table 2 we observed the distribution of the participants classified by the quartiles of narrative creativity (low, medium and high) and NFC quartiles (low, medium and high), excluding the lie scale group.

To study the interrelation between narrative creativity and need for cognitive closure, we applied the chi-square (χ^2^) and Pearson’s correlation tests. Statistical χ^2^ gives us the result *p* = 0.016, therefore, it can be concluded that NC and NFC are significantly related. Applying Pearson’s correlation, the value of r = −0.26, the correlation is statistically significant (*p* = 0.19), that is to say NC and NFC show a low negative correlation.

It was detected that neither the correlation between the final note and total result in NFC, nor the correlation between the final note and any of the 5 NFC factors were significant (r of Pearson; *p* > 0.05 for all the cases).

### 3.3. Lie Scale Group Analysis

As 34 of 117 participants (29.06%) were discarded based on the lie scale (lie scale higher than 15), we considered this percentage too high and supposed that if we analyzed the lie scale group with the valid answers group, we may find differences regarding the gender, school type, group belonging or narrative creativity variables.

First of all, in the lie scale group we found 21 male students (42% of the total in the lie scale group) (Table 3). The corresponding proportion for females is 19.40%. The difference is notable that shows female participants were sincere (or motivated) to answer the test.

As for the differences regarding the school type, we found that the percentage of the lie scale group was higher in the rural area school (36%) than in the urban area school (23.88%).

We observed (Table 4) that there were two groups in the rural public schools where the percentage of the lie scale group was high: group 1RUR (53.33%) and 3RUR (41.67%). As a description of the group, it should be noted that both groups in this school had the same English teacher, while the group 2RUR had a different English teacher. We can assume that some factors interact within these groups that we cannot detect in this study and lower the motivation of the students, which was reflected in the lying scale in one of the applied instruments.

### 3.4. Lie Scale and Narrative Creativity

We observed that the average index of narrative creativity among the lie scale group of students is notably lower than in the group of sincere students. After applying a t-test to an independent sample we detected that the difference between the mean values of 52.98 and 39, taking into account their corresponding standard deviations, is statistically significant at the population level (*p* = 0.004). This shows that non lie scale group students seem to have more creativity than those who belong to the lie scale group (although the ratio between both groups is not unequal, 83 compared to 34).

### 3.5. Lie Scale and Final Note

It was observed that the average note in the lie scale group is lower than in the non lie scale group. When applying the t-test to independent samples we found a statistically significant difference (7.17 for non lie scale group and 6.07 for the lie scale group, Table 5). It showed that those students in the lie scale group have a lower academic achievement than students who filled the test in properly.

## 4. Discussion

First of all, we detected that narrative creativity is correlated with two of the five factors of need for cognitive closure: order and predictability. This concurs with the results obtained in the study of García-Ramírez and Hazir [3]. Their results focus on university level students; in our case we detected the same relationship in adolescents aged 14–16 years. Additionally, we can see that narrative creativity was extracted from the graphic task, confirming the correlation mentioned, not only general creativity (the sum of narrative and figurative tasks).

Regarding the general NFC score, NC and NFC seem to show a low negative correlation which also proves the results obtained in the studies of Rocchi [1], Chirumbolo et al. [2], García-Ramírez and Hazir [3], García-Ramírez [4] and Sankaran et al. [5]. Again, in the example of narrative creativity, it seems to be correlated with NFC in the same way as general creativity.

At the same time, we pointed out that both correlations seem to be present in secondary education students (in our case, youngsters of 14–16 years old), not only in adults or university students as was detected in the antecedent studies.

Secondly, another aim of our study was to analyze the group of students who entered into the lie scale group integrated into the Need for Closure Scale. These participants could not be analyzed for the correlation of NFC and narrative creativity, but they were analyzed as a separate group to find a possible correlation with the rest of the variables. The statistically significant correlation between the lie scale group was found for all of the variables: gender, group, narrative creativity and the English as a foreign language course final note. Although the lie scale group participants and non lie scale participants proportion were not equal, the findings show the interest of analyzing the lie scale group in the studies where more tools rather than the Need for Closure Scale are applied.

All of these correlations may take root in the pure will of lying as the results show low levels of motivation in realizing the task. As demonstrated by Eysenck et al. [20], a test realization may depend on a high grade of motivation by the student when realizing the task. On the other hand, it can be related to the broader origin of the motivation to perform tasks in class as the test was passed during the English as a foreign language classes. In the context of the present study, we did not have background information about the participants at the individual level, but it was necessary to establish the possible relationship between motivation and the lie scale results. Alternatively, the personality test including the honesty variable and NFC indexes, taking into account the lie scale group, could be realized with the aim of establishing the origin of the elevated number of invalid results when applying the Need for Closure Scale.

Regarding gender, we need to highlight that in the few similar studies in the field of NFC and creativity [1,2,3,4,5], the results are not interpreted following gender division. In the present study, male participants showed a tendency to give more invalid answers, which can be related with the personal gender differences and reflective of the attitude of motivation/demotivation or being sincere/insincere when answering the test questions. These personality differences may also depend on the area where students live and study. The results of our study show that in rural areas the percentage of lie scale participants is higher than in the urban area school. The differences also arise at a group level; several groups can be distinguished based on the elevated percentage of invalid answers. It is notable that in the rural area, two of the rural area groups had the same English teacher and that both of these groups have higher numbers compared with other groups. This brings us back to the hypothesis regarding the motivational aspects in an educational context that may foster low motivation for realizing the task.

Finally, we found a statistically significant correlation between narrative creativity and the final note. The participants in the lie scale group had lower course notes and lower creativity. This sustains a hypothesis that the reasons of belonging to the lie scale group are based on the low motivation of the students rather than their personal character features, for example, dishonesty.

Most of the results obtained point out that creativity and divergent thinking can be hampered by cognitive closure, whose origin is in intergroup relations with the intervention of social pressure [4]. Therefore, it is important to monitor cognitive closure from a very early age. If García Ramírez’s studies have revealed the same results with university level students, when working with secondary school students, we have observed that factors of cognitive closure are already involved in this educational stage and must be controlled in order to prevent the negative consequences related to the need for cognitive closure.

This study has several limitations that must be considered. The first is related to the sample size. Another point that could be mentioned is the scarcity of previous studies on narrative creativity and the need for cognitive closure. Additionally, there are limitations related to the instruments used. The Test of Creative Imagination for Young People is a qualitative tool that requires a long time to obtain the score from each questionnaire. Finally, it was difficult to predict the proportions of the lie scale group and non lie scale group to achieve more equality between groups. The differences found in the present study should be confirmed by amplifying the sample in future studies.

The results found in the present study contribute to the field of developmental psychology and point out the need for further research of the need for cognitive closure and creativity not only in adults, but also in adolescents.

## 5. Conclusions

As for the first objective, the results of the present research point out a negative correlation between narrative creativity and the need for cognitive closure in adolescents. Here the need for cognitive closure is mainly shown in two of five dimensions: order and predictability.

As for the second objective, we found a high number of students in the lie scale group integrated into the Need for Cognitive Closure Scale. These students were analyzed as a subgroup in comparison with sincere students. The analysis revealed that male students tend to not answer sincerely more often. At the same time, the percentage of lie scale group participants tended to be higher in the rural area school. Finally, students not belonging to the lie scale group seem to have more creativity than lie scale group students, while lie scale group students seem to have a lower final note on their course than non lie scale group students.

## Figures and Tables

**Table 1:** Average scores for the five factors of the test for the need for cognitive closure in the participants (with the exception of those exceeding 15 on the lie scale) classified according to the quartiles of narrative creativity and gender.

NC	Order		Predictability		Decisiveness		Ambiguity		Closed-Mindedness	
	M	F	M	F	M	F	M	F	M	F
Low	41.99	42.08	28.56	29.37	27.54	23.33	38.82	37.85	23.9	22.49
Medium	44.3	39.88	28.55	29.56	26.65	24.73	39.5	37.88	21.6	22.37
High	33	34	26.33	25.38	20.67	23.17	34.67	38.46	22.33	20.75
F	18.20		5.93		2.05		9.03		1.50	
*Sig.*	0.000 *		0.004 *		0.134		0.089		0.229	

Note: Narrative Creativity (NC). * Statistical differences at level *p* < 0.05.

**Table 2:** Contingency table showing the number of participants classified by levels of narrative creativity and the need for cognitive closure.

Narrative Creativity	Need for Cognitive Closure			Total
	Low (Q1)	Medium (Q2)	High (Q3)	
Low (Q1)	5	10	12	27
Medium (Q2)	6	23	9	38
High (Q3)	9	5	4	18
Total	20	38	25	83

**Table 3:** Percentage of lie scale group and non lie scale group regarding gender and school type.

Variable	Male	Female	Urban School	Rural School
Non Lie Scale	29 (58%)	54 (80.6%)	51 (76.12%)	32 (64%)
Lie Scale	21 (42%)	13 (19.4%)	16 (23.88%)	18 (36%)
*N*	50	67	67	50

**Table 4:** Percentage of lie scale group and non lie scale group regarding group distribution.

Variable	1RUR	2RUR	3RUR	4URB	5URB	6URB	*N*
Non-Lie Scale	7 (46.67%)	18 (78.26%)	7 (58.33%)	20 (83.33%)	16 (66.67%)	15 (78.95%)	83
Lie Scale	8 (53.33%)	5 (21.74)	5 (41.67)	4 (16.67%)	8 (33.33%)	4 (21.05%)	34
*N*	15	23	12	24	24	19	117

Note: Rural (RUR); Urban (URB).

**Table 5:** Correlation of lie scale group and non lie scale group with narrative creativity and final note.

Variable	*N*	NC	Final Note
		M (SD)	M (SD)
Non Lie Scale	83	52.98 (24.47)	7.17 (1.81)
Lie Scale	34	39 (19.09)	6.07 (2.22)

Note: Narrative Creativity (NC).

## Data Availability

The data presented in this study are available on request from the corresponding author.
